# An interdisciplinary approach for the management of periapical lesion using regenerative approach: a case report

**DOI:** 10.1186/s13256-025-05380-2

**Published:** 2025-07-09

**Authors:** Priyanka Jaiswal, Sakshi Vishal Kotecha, Shweta Bhagat, Shweta Sedani, Akash Thakare

**Affiliations:** 1 Department of Periodontics and Implantology, Sharad Pawar Dental College, Datta Meghe Institute of Higher Education and Research, Sawangi, Meghe, Wardha, Maharashtra India; 2 Department of Conservative Dentistry and Endodontics, Sharad Pawar Dental College, Datta Meghe Institute of Higher Education and Research, Sawangi, Meghe, Wardha, Maharashtra India

**Keywords:** Periapical lesions, Pus discharge, Periapical radiolucency, Dental cyst, Multidisciplinary approach, Case report

## Abstract

**Background:**

Periapical lesions are common sequelae of endodontic infections, often presenting as chronic apical periodontitis. These lesions result from the microbial invasion of the root canal system, leading to inflammation and subsequent bone resorption. While conventional root canal therapy is the primary treatment modality, it occasionally fails to resolve periapical pathology. In such cases, surgical intervention becomes necessary.

**Case presentation:**

This report presents the case of a 32-year-old female of Indian ethnicity with chief complaints of pain and pus discharge in the upper right front area in the past 2 months. Radiographic findings were evident, depicting periapical radiolucency. Upon debriding the lesion, it was observed to be creamish with irregular shape and size and, hence, it was sent for histopathological analysis. It was inferred to be a dental cyst associated with the right upper front region of the jaw.

**Conclusion:**

This case report pinpoints the importance of considering the diagnosis of periapical lesions. It also adds to the point of treatment plan that can be followed as a multidisciplinary approach to challenging periapical lesions.

## Introduction

Periapical or periradicular lesions are barriers that restrict the microorganisms and prevent their spread into the surrounding tissues [1]. The majority of periradicular lesions can be categorized as dental granulomas, periradicular cysts, or abscesses, which are radiolucent [2]. The likelihood of periradicular cyst is much higher in the presence of the following conditions: (a) the periradicular lesion involving one or more teeth with necrotic pulps; (b) the lesion being ≥ 200 mm^2^; (c) aspiration yielding a straw-colored fluid or drainage of such fluid through an access; and (d) the presence of cholesterol crystals in the fluid [3]. Furthermore, the incidence of cysts has been reported to be 60–67% in lesions measuring 10–20 mm in diameter [4]. When considering the lesion volume, there is an 80% probability of a cyst if it measures > 247 mm^3^ and a 60% probability with root displacement and a volume < 247 mm^3^ [5]. Numerous treatment options have been employed for the treatment of large periradicular lesions, ranging from root canal therapy to different surgical interventions. Surgical approaches may lead to various consequences, such as possible damage to the adjacent vital teeth, damage to the anatomic structures in the vicinity of the lesion, and pain and discomfort, whereas in the case of nonsurgical approaches, the treatment is less invasive with better patient acceptance [6].

Restoring lost periodontal tissue is the major objective of periodontal therapy to preserve the health and functionality of teeth. “Creating new alveolar bone, cementum, and periodontal ligament on a previously damaged root surface is known as periodontal tissue regeneration [7].” Numerous therapeutic approaches have been investigated for periodontal tissue regeneration [7].

Numerous regenerative periodontal therapies have been developed over the past 20 years with the goal of replacing lost tooth-supporting tissues, such as “the cementum, alveolar bone, and periodontal ligament” [8]. The various regenerative materials used are guided tissue regeneration (GTR), enamel matrix derivative, and bone grafts such as autografts, allografts, and xenografts [9].

The primary function of bone substitutes is to serve as a structural framework for the correct sequence of biological processes [10]. Among calcium ceramics, hydroxyapatite, β-tricalcium phosphate, and biphasic calcium phosphate [11] are widely used owing to their similarity to bone minerals, excellent biocompatibility, and ease of handling. These materials promote natural degradation and absorption, facilitating the growth of new bone tissue [12]. Tricalcium phosphate (TCP) is also in use, particularly when combined with hydroxyapatite (HA), which exhibits additional desirable characteristics such as restorability and osteo-conductivity [13].

In the treatment of osseous abnormalities, platelet-rich plasma (PRP) has been used to improve the clinical results achieved with bone grafts both with and without directed tissue regeneration. However, using PRP may come with some hazards [14]. The second-generation platelet concentrate, platelet-rich fibrin (PRF), was developed by Choukroun and colleagues in 2001. It offers several advantages over traditional platelet-rich plasma (PRP). PRF was designed to optimize growth hormone release and reduce hypersensitivity by removing anticoagulants. Leukocytes and platelets are encased in a fibrin matrix produced by the platelet-rich layer. Unlike PRP, this matrix permits growth factors to be released gradually and continuously [15]. Because PRF can reduce osteoclastogenesis by encouraging the release of osteoprotegerin (OPG) in osteoblast cells and upregulate the expression of phosphorylated extracellular signal-regulated protein kinase, it may facilitate the healing of periodontal osseous defects [16]. In addition, it has been demonstrated that PRF increases the expression of OPG and alkaline phosphatase (ALP), which support the osteogenic differentiation of human dental pulp cells [17].

The report emphasizes that there is a notable connection between oral bacterial invaders and the body’s defensive mechanism at the tooth apex, leading to periapical lesions and facilitating the spread of infections, which are often asymptomatic until they present acute signs of inflammation or discomfort. This underscores the importance of early diagnosis and intervention in managing such conditions. Periapical lesions pose a diagnostic challenge. The difficulty in making a differential diagnosis complicates the prognosis for the affected teeth. In this specific case, the patient presented with pain and pus discharge, and radiographic findings indicated periapical radiolucency. This suggests that the infection may have originated from the pulp, highlighting the need for a thorough examination to determine the source of the problem. This case report also advocates for a multidisciplinary treatment plan when addressing periapical lesions. This approach is essential for improving the prognosis of the affected teeth, as it allows for comprehensive management of both the endodontic and periodontal aspects of the condition.

Thus, in the following case, the regenerative potential and healing span was evaluated with the use of PRF and β-tricalcium phosphate in the combined periapical lesions.

## Case report

**Fig. 1:**
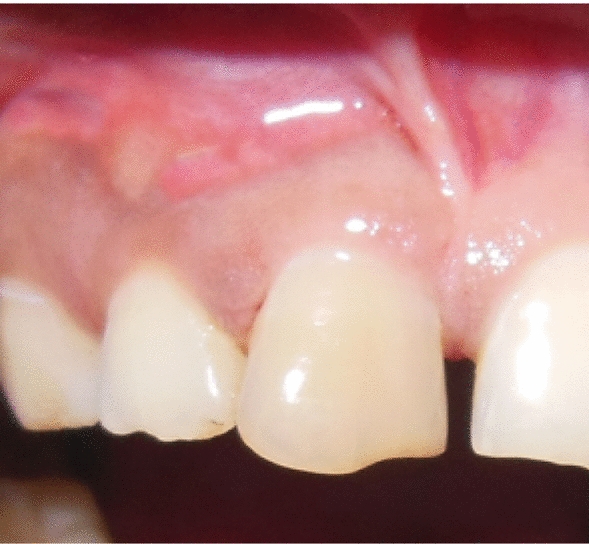
Pus discharge and swelling in upper anterior region of the jaw

**Fig. 2:**
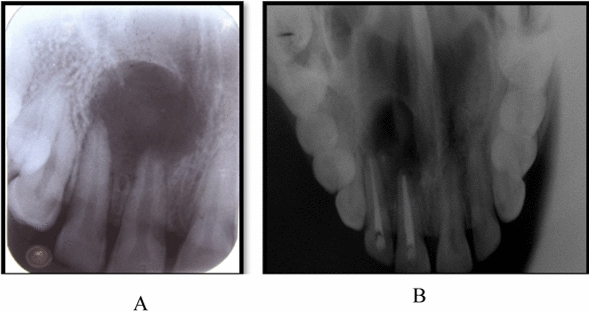
**A** Periapical radiolucency was seen on intraoral periapical radiograph associated with regions 11 and 12. **B** Periapical radiolucency was seen on occlusal radiograph associated with regions 11 and 12

A 32-year-old woman of Indian ethnicity without any prior health issues and family history presented with pain and pus discharge in the upper right front area of the jaw for the past 2 months (Fig. 1). The patient experienced trauma in the anterior region 1 year prior. She complained of pain and pus discharge within the last 2 months. Teeth 11 and 12 were tender on percussion, and tooth 11 was discolored. Radiographic examination revealed periapical radiolucency of diameter more than 10 mm with tooth 11, with tooth 12 extending from the mesial border of tooth 21 to the distal border of tooth 12 (Fig. 2). A preliminary clinical diagnosis of periapical cyst was made on the basis of the following factors:

1. Anterior teeth were affected by the periapical lesion.
2. The lesion appeared on radiography as a well-defined, bounded, radiolucent region with a thin radiopaque line.

**Fig. 3:**
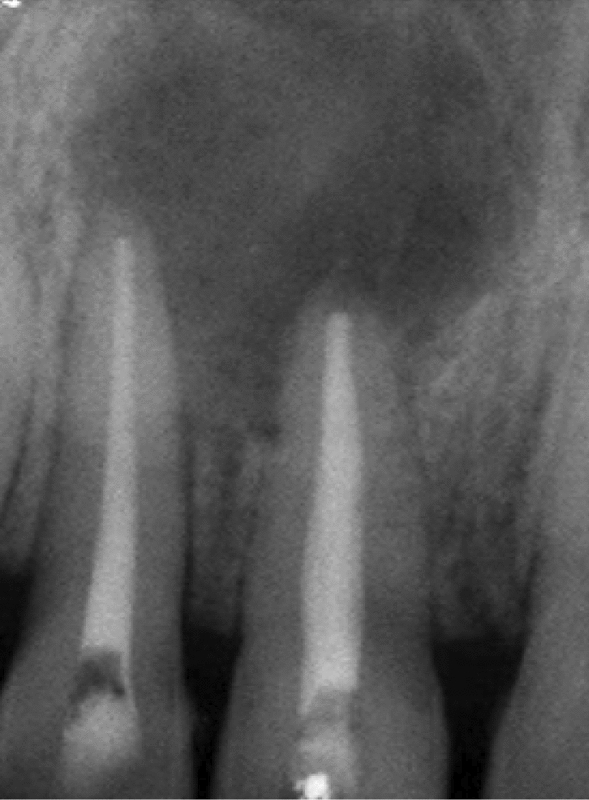
Endodontical treatment was performed on regions 11 and 12

The primary goal of treating teeth 11 and 12 with root canal therapy was to entirely eradicate these bacteria. The patient signed an informed consent form. Local anesthesia was administered. Canals were found, and an access opening was made. In the following appointments, protaper (Dentsply) was used to finish the root canal treatment, and saline and 5.25% sodium hypochlorite were used for irrigation. The cold lateral compaction procedure was used to obturate the teeth (Fig. 3). Following the endodontic phase, the patient still complained of pain in the upper anterior region, with persistent presence of pus discharge in the region of teeth 11 and 12. Therefore, it was decided to treat the present case with an apicoectomy procedure to gain access to the periapical lesion, followed by osseous regenerative procedure using autologous platelet rich fibrin (PRF) + bone graft.

**Fig. 4:**
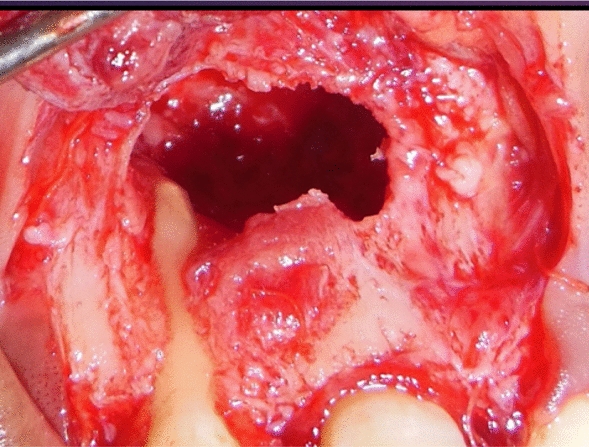
Full thickness mucoperiosteal flap was reflected 3 mm apical to the lesion

**Fig. 5:**
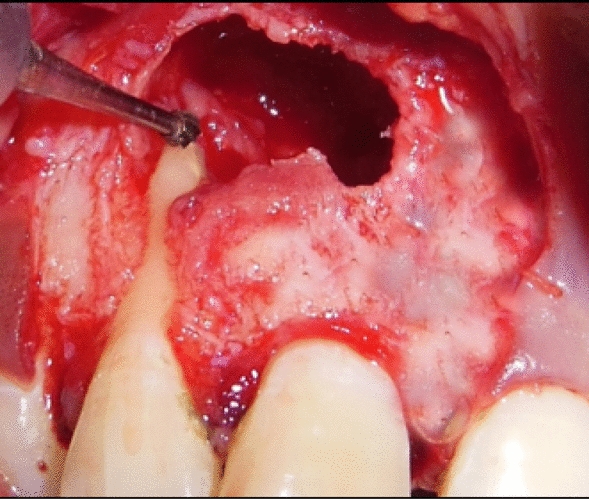
Carbide bur was used to prepare and resect the apical third of the root portion

**Fig. 6:**
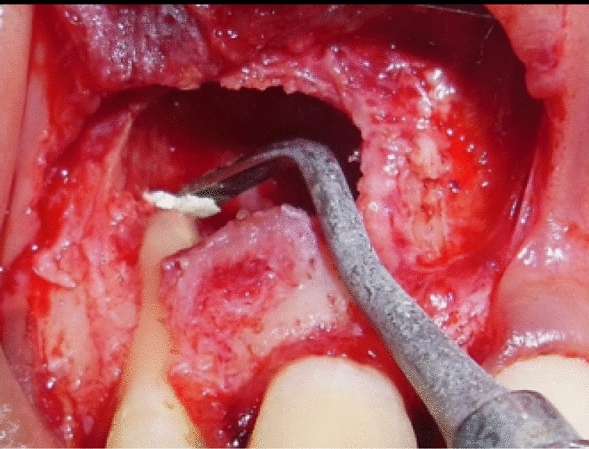
Mineral trioxide aggregate was placed after root resection and complete debridement of lesion

Under all aseptic conditions and precautions, local anesthesia was administered, and full thickness mucoperiosteal flap was reflected 3 mm apical to the lesion (Fig. 4). Debridement of the lesion was carried out with curettes, and tissue biopsy was obtained from the base of the defect close to the root apex for histopathological evaluation. Root end preparation was carried out, the apical third of the root portion was resected using a round carbide bur (Fig. 5), and mineral trioxide aggregate (MTA) was placed (Fig. 6). For PRF preparation, 5 ml of blood was withdrawn from antecubital fossa and centrifuged at 3000 rpm for 10 minutes, and sterile forceps and scissors were used to separate the PRF from the red blood cells.

**Fig. 7:**
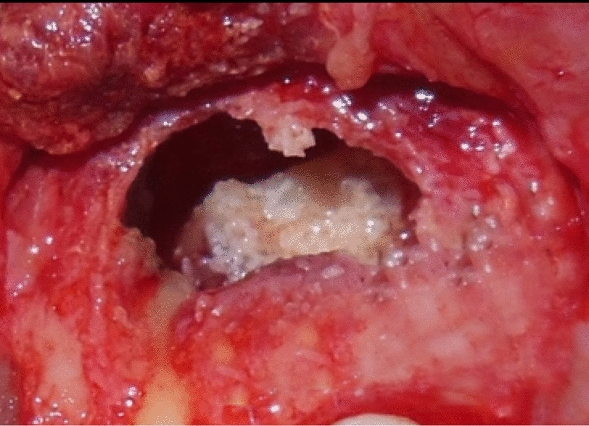
Placement of β-tricalcium phosphate and hydroxyapatite mixed with platelet-rich fibrin

**Fig. 8:**
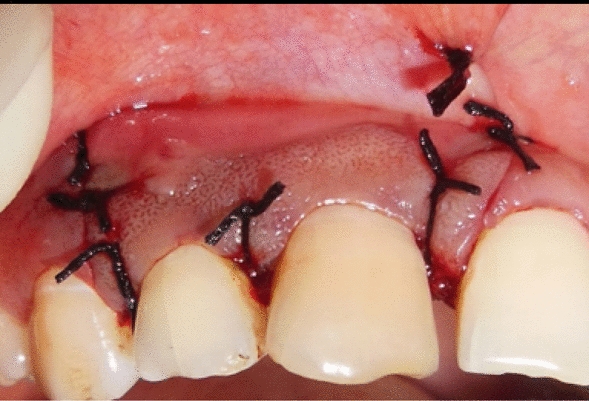
Flap approximation performed using simple interrupted sutures

Autologous PRF mixed with β-TCP HA was then placed into the defect, and flap was approximated by using simple interrupted sutures (Figs. 7 and 8, respectively).

**Fig. 9:**
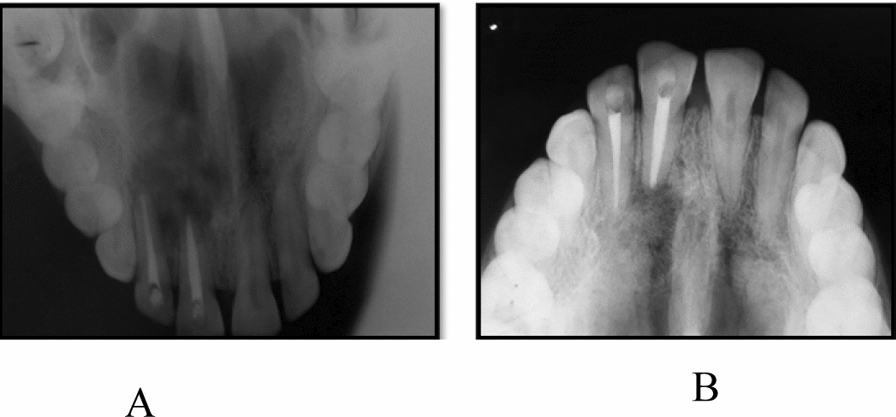
**A** Radiograph taken after 3 months. **B** Radiograph taken after 6 months

Patient was followed-up after 1 week, 3 months, and 6 months (Fig. 9A, B)

**Fig. 10:**
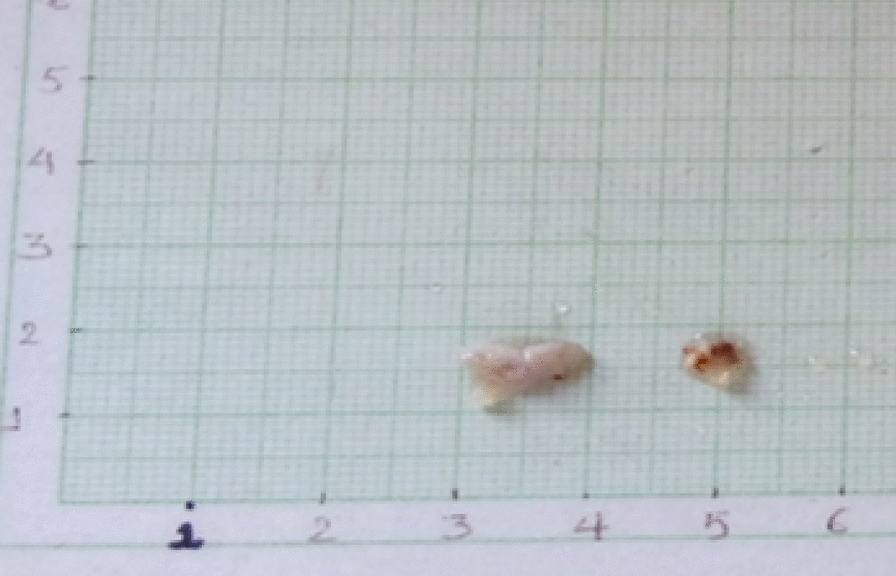
Image depicts the color and size of lesion

**Fig. 11:**
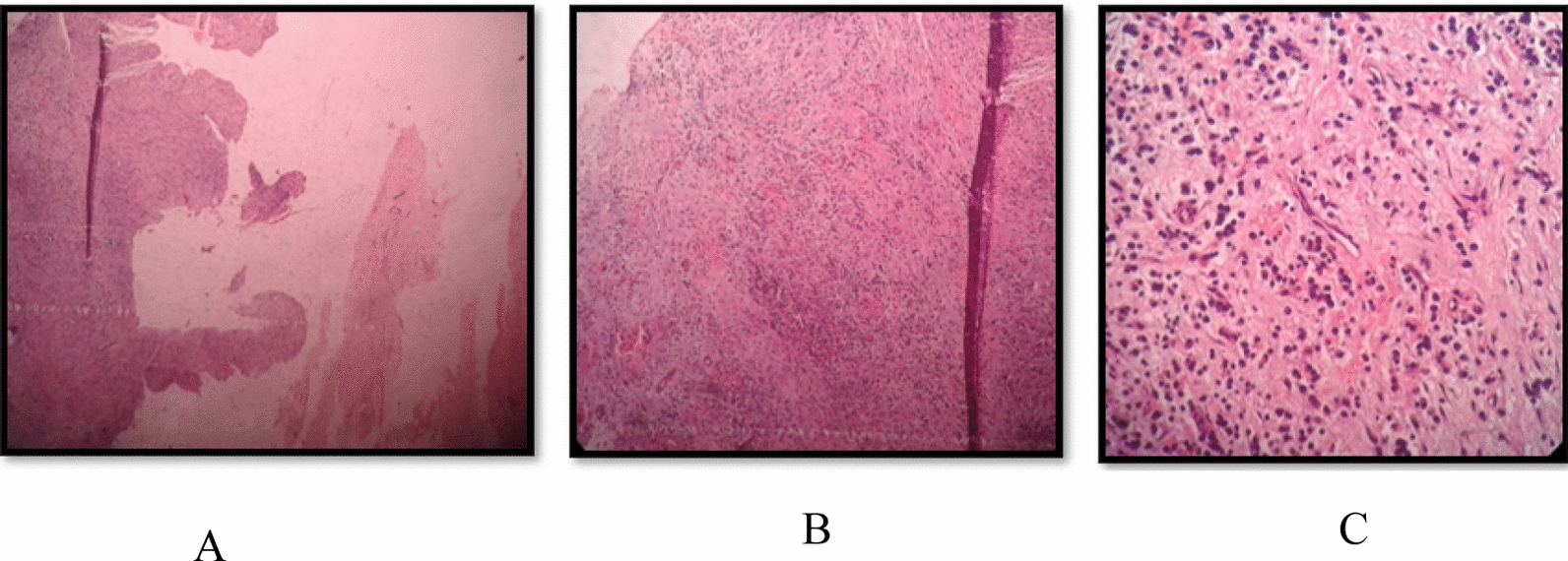
**A** Specimen seen under microscope at 4×. **B** Specimen seen under microscope at 10×. **C** Specimen seen under 40×

Upon histopathological examination, it was observed that the size was 1 cm × 1 cm × 1 cm (approximately) with irregular shape and surface. The color appeared to be creamish (Fig. 10). The specimen was observed under 4×, 10×, and 40× (Fig. 11A–C). Histopathology report confirmed it to be an infected radicular cyst.

Postoperatively, no complications were observed, and clinically, healing was uneventful at 1 week postsurgery. Gingiva on labial surfaces of teeth 11 and 12 clinically appeared to be healthy with disappearance of sinus opening. At 3 months postsurgery, radiograph showed 60–70% increase in radio-opacity of the lesion, and at 6 months, radiograph showed 90–95% increase in radio-opacity.

## Discussion

The present case demonstrates the therapeutic success of β-TCP and hydroxyapatite and PRF in its capacity to generate bone structure. The unpredictability of periapical lesion treatment stems from the diagnostic challenges it poses. Nevertheless, a thorough patient history and meticulous examination can lead to a definitive diagnosis, paving the way for a successful treatment plan [18].

The most important principle of treating periradicular lesions is to eliminate the bacteria. A large periradicular lesion may have direct communication with the root canal system and respond favorably to nonsurgical treatment [19]. Large periapical lesions are of inflammatory origin, as well as apical true cysts, and should be treated initially with a nonsurgical approach. When intra- or extraradicular infections are persistent, and periapical pathology fails to resolve after nonsurgical endodontic management protocols, only then should a surgical option be considered [20].

The emergence of tissue engineering and growth factors has renewed optimism for the regeneration of lost periodontal tissues. PRF and PRP, enriched in growth factors such as transforming growth factor (TGF)-beta and platelet-derived growth factor (PDGF), exhibit angiogenic, proliferative, and osteogenic properties, fostering bone healing [13].

PRF and PRP have shown efficacy when used in conjunction with different graft materials to address periodontal defects [21].

In their investigation, Taschieri *et al.* combined xenogenic bone grafts with autologous growth factors to treat through-and-through bone lesions. This accelerated and demonstrated consistent tissue regeneration [22].

Jayalakshmi and her team reported a case where PRF and β-TCP bone graft were successfully used to promote rapid healing of a periapical lesion. Regular follow-ups at 3, 6, 9, and 12 months showed smooth healing with no complications. Subsequent evaluations consistently demonstrated significant and predictable bone regeneration both clinically and radiographically, without any adverse effects. In contrast to employing these biomaterials separately, the study found that combining PRF and β-TCP for bone augmentation in periapical defects could be a potential therapy option for quicker healing [23].

A similar case report by Kavitha *et al.* suggested that the therapeutic outcome was encouraging, by using PRF with β-TCP. According to the authors, for large periapical lesions, platelet concentrates such as PRF and PRP mixed with β-TCP may be a good substitute for conventional bone grafts and membranes. This combination could potentially accelerate bone regeneration and reduce healing time [24].

PRF combined with β-tricalcium phosphate bone graft not only promotes bone growth, maturation, and wound healing but also provides improved handling characteristics, wound sealing, hemostasis, and graft stabilization [25].

### Strengths

1. The report emphasizes the importance of accurately diagnosing periapical lesions, which can often be challenging owing to their complex nature. By highlighting this aspect, the report aids clinicians in recognizing and addressing these conditions effectively.
2. It advocates for a multidisciplinary treatment plan, which is crucial for managing challenging cases such as periapical lesions. This approach can lead to better patient outcomes by integrating various specialties in dental care.
3. The case report provides a thorough presentation of the patient’s history, symptoms, and clinical findings. This detailed account allows readers to understand the progression of the condition and the rationale behind the treatment decisions made.
4. The report includes histopathological findings that confirm the diagnosis of a dental cyst. This adds a layer of scientific rigor to the case, as it supports the clinical diagnosis with laboratory evidence.
5. The inclusion of radiographic examinations showing periapical radiolucency provides visual evidence of the condition, which is essential for understanding the extent of the lesion and planning appropriate treatment.
6. The report suggests the need for further clinical trials to explore the effectiveness of using platelet derivatives in treating periapical lesions. This opens avenues for future research and could lead to advancements in treatment protocols.
7. The discussion references similar cases where the use of platelet-rich fibrin (PRF) combined with β-TCP showed promising results. This not only supports the findings of the current case but also encourages further exploration of innovative treatment options.

### Limitations

1. The report is based on a single case, which limits the generalizability of the findings. Results from one patient may not be applicable to a broader population, making it difficult to draw definitive conclusions about the effectiveness of the treatment approach used.
2. There is no mention of long-term follow-up data to assess the durability of the treatment outcomes. Without this information, it is challenging to evaluate the long-term success and potential complications associated with the treatment.
3. The case report does not include a control group for comparison, which is essential for establishing the efficacy of the treatment. Without a control group, it is difficult to determine whether the observed outcomes were due to the treatment or other factors.
4. The findings are based on a single patient, which does not provide sufficient statistical power to support the conclusions drawn. Larger studies with more participants are needed to validate the results and provide a more comprehensive understanding of the treatment’s effectiveness.
5. The report highlights the necessity for more carefully monitored clinical trials over a longer time frame to determine the true impact of using platelet derivatives in combination with bone grafts. This indicates that the current evidence is not yet robust enough to support widespread clinical application.

## Conclusion

To determine whether the use of platelet derivatives, either by themselves or in combination with bone grafts, considerably improves bone repair in periapical lesions, more carefully monitored clinical trials over a longer time frame are needed. Using PRF, as explained, allows the clinician to utilize its complete regenerative potential.

## Data Availability

Not applicable.
